# Beneficial impact of intensified multifactorial intervention on risk of stroke: outcome of 21 years of follow-up in the randomised Steno-2 Study

**DOI:** 10.1007/s00125-019-4920-3

**Published:** 2019-06-01

**Authors:** Peter Gæde, Jens Oellgaard, Christina Kruuse, Peter Rossing, Hans-Henrik Parving, Oluf Pedersen

**Affiliations:** 1 Department of Cardiology and Endocrinology, Slagelse Hospital, Slagelse, Denmark; 2 Institute for Regional Health Research, University of Southern Denmark, Odense, Denmark; 3 Novo Nordisk Scandinavia A/B, Region Denmark, Ørestad, Denmark; 4 Department of Neurology, Herlev Gentofte Hospital, University of Copenhagen, Herlev, Denmark; 5 Steno Diabetes Center Copenhagen, Gentofte, Denmark; 6 Faculty of Health Sciences, University of Copenhagen, Copenhagen, Denmark; 7 Department of Endocrinology, Rigshospitalet, Copenhagen, Denmark; 8 Novo Nordisk Foundation Center for Basic Metabolic Research, Section of Metabolic Genetics, Faculty of Health and Medical Sciences, University of Copenhagen, Panum, Maersk Tower, Blegdamsvej 3B, 2200 Copenhagen N, Denmark

**Keywords:** Microalbuminuria, Multifactorial intervention, Stroke, Type 2 diabetes

## Abstract

**Aims/hypothesis:**

Epidemiological studies have shown that diabetes is a well-established independent but modifiable risk factor for stroke. The aim of this post hoc analysis of data from the Steno-2 Study was to examine whether multiple risk factor intervention reduced the risk for stroke in individuals with type 2 diabetes and microalbuminuria.

**Methods:**

In the Steno-2 Study, 160 individuals with type 2 diabetes and microalbuminuria were randomised to intensified or conventional multiple risk factor intervention, targeting classical cardiovascular disease risk factors for a mean of 7.8 years, and then followed for a total mean of 21.2 years. The primary endpoint in this post hoc analysis was time to first stroke event.

**Results:**

During follow-up, 30 participants experienced a total of 39 strokes. Individuals randomised to conventional therapy were more likely to experience a stroke than those in the intensive-therapy group, with 29 total strokes occurring in 21 participants (26%) in the conventional-therapy group vs a total of ten strokes in nine participants (11%) in the intensive-therapy group (HR 0.31 [95% CI 0.14, 0.69]; *p* = 0.004). Also, the number of recurrent strokes was significantly reduced with intensive therapy.

**Conclusions/interpretation:**

Intensified multiple risk factor intervention in patients with type 2 diabetes and microalbuminuria reduces the risk for strokes as well as the number of recurrent cerebrovascular events.

**Trial registration:**

ClinicalTrials.gov NCT00320008.

**Electronic supplementary material:**

The online version of this article (10.1007/s00125-019-4920-3) contains peer-reviewed but unedited supplementary material, which is available to authorised users.

## Introduction

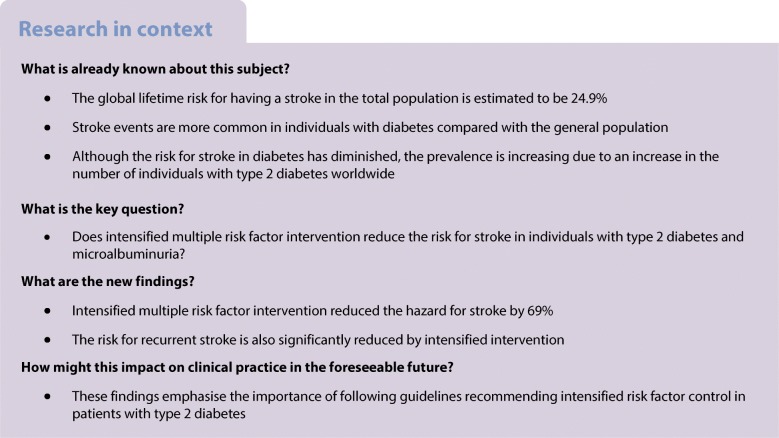



A recent publication from the Global Burden of Disease (GBD) Study 2016 estimates the global lifetime risk of stroke to be 24.9% in the total population for both sexes, with an increase in risk of 15.4% for men and 3.2% for women compared with the previous survey, which was conducted in 1990 [1].

In individuals with diabetes in the USA, an increased incidence of stroke from 1990 to 2010 has been demonstrated. Although a 52.7% decrease in the age-standardised rates of stroke was seen, the increase in diabetes prevalence resulted in a 47% increase in the yearly number of strokes [2].

The importance of individualised intensified multifactorial intervention in the treatment of type 2 diabetes has been highlighted in the Steno-2 Study. In this study, an increased median lifespan of 7.9 years with significant risk reductions for major cardiovascular disease (CVD) events, heart failure and microvascular complications was observed with intensified treatment of multiple risk factors vs conventional therapy [3]. Also, it has been demonstrated that these results were achieved at no extra cost; the total direct cost of the intensified multifactorial intervention was similar to that of conventional treatment [4].

In the present post hoc analysis, the primary aim was to examine the difference in time to first stroke event between the intensive and conventional treatment groups of the Steno-2 Study.

## Methods

### Study design

**Fig. 1:**
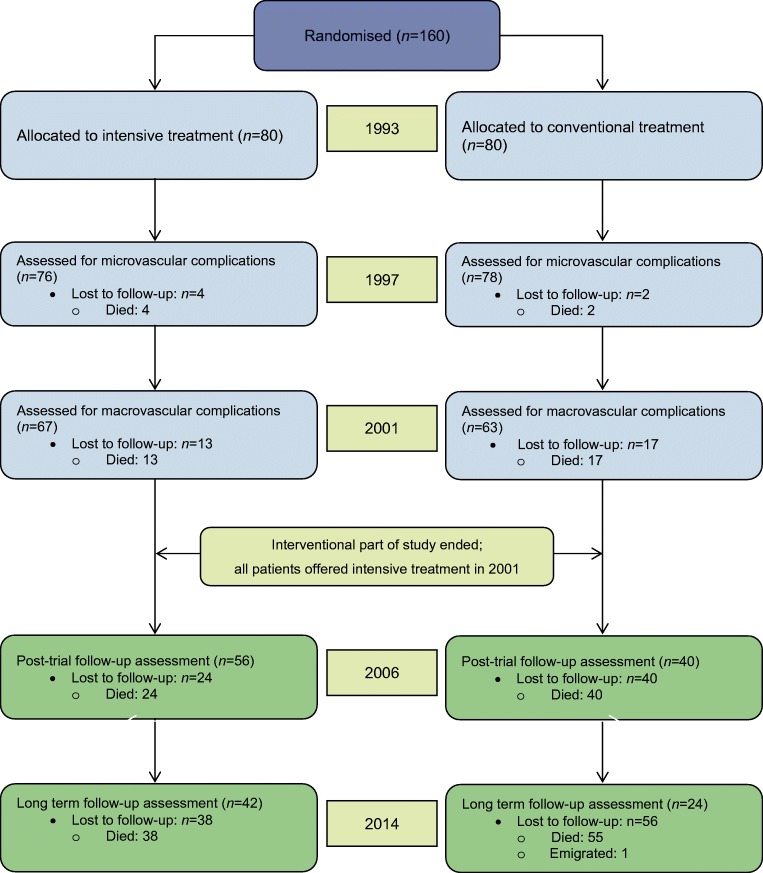
CONSORT diagram showing participant flow throughout the Steno-2 trial. The first 7.8 years were the active intervention period, after which time the randomisation was neutralised and continued as a post-trial observational follow-up study with all remaining participants being offered the same treatment as the original intensive-therapy group. At the time of randomisation, the mean age was 55.1 years and 66% were male. All participants had microalbuminuria. Reproduced from [8] under the terms of the Creative Commons Attribution 4.0 International License (http://creativecommons.org/licenses/by/4.0/), which permits unrestricted use, distribution and reproduction in any medium

The Steno-2 Study (ClinicalTrials.gov registration no. NCT00320008) has been described in detail previously [3–5]. In short, during 1992 and 1993, 160 participants with type 2 diabetes and microalbuminuria were randomised to conventional or intensified multifactorial treatment targeting several concomitant risk factors (*n* = 80 in each group; Fig. 1). Mean treatment duration was 7.8 years. After 7.8 years, the randomised part of the study ended and all individuals were offered intensified multifactorial treatment, allowing the trial to continue as an observational follow-up study for an additional 13.4 years.

The protocol for the follow-up trial in the Steno-2 Study was conducted in accordance with the declaration of Helsinki and approved by a local ethics committee (Ethics committee, Capital Region of Denmark; protocol ID number: H-KA-99035-GS, add. 41104) and by the Danish Data Protection Agency (J.Nr. 2015-41-4042). All patients gave their informed consent upon randomisation and confirmed this on follow-up visits.

### Endpoint definitions

All CVD endpoints in the Steno-2 Study were defined a priory and have been described in detail elsewhere [5]. The definitions for stroke and transient ischaemic attack (TIA) are shown in the electronic supplementary materials (ESM) Methods. All possible cases of stroke and TIA were adjudicated by an external committee masked for original treatment allocation.

In the current analysis, we distinguish between stroke and TIA. This was not the case in the original Steno-2 Study where we used a combination of the two conditions in order to increase power due to the shorter duration of follow-up and, thus, a smaller number of expected events.

The primary analysis of the present study was time to first stroke event, with the secondary analyses being time to a combined endpoint of stroke and cardiovascular death and time to a combined endpoint of stroke and all-cause mortality. In sensitivity analyses, cases of TIA were added to the endpoints specified above.

### Statistical analyses

HRs for the primary and secondary outcomes of this post hoc analysis were calculated using Cox regression. Recurrent strokes were compared using Fisher’s exact test. Statistical analyses were performed using Stata/IC version 15 (StataCorp, College Station, TX, USA). For the primary outcome, proportional hazards assumption was checked by the ‘estat phtest’ function of Stata.

## Results

Baseline values in each of the two original treatment groups are shown in the ESM Table 1. During a follow-up of 21.2 years, 30 individuals experienced a total of 39 stroke events. Participants randomised to conventional therapy were more likely to experience a stroke, with a total of 29 strokes occurring in 21 individuals (26%) in the conventional-therapy group, compared with a total of ten stroke events in nine individuals (11%) in the intensive-therapy group.

**Fig. 2:**
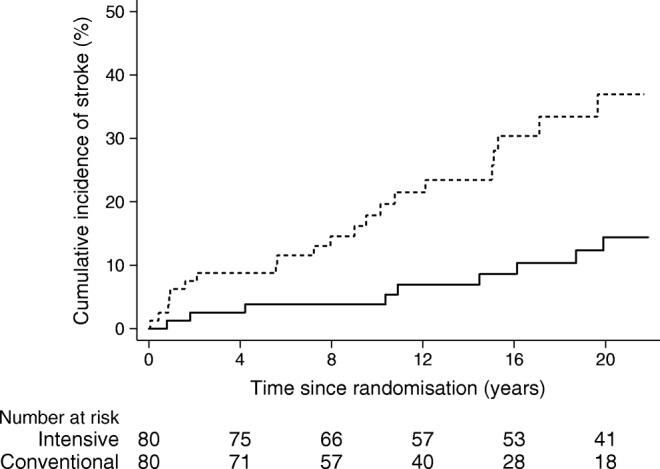
Cumulative incidence frequency plot of time to first stroke. The hazard for stroke was reduced by 69% in the intensive-therapy group (*p*=0.004). Solid line, intensive-therapy group; dashed line, conventional-therapy group

The hazard for stroke was reduced by 69% with intensive therapy vs conventional treatment (HR 0.31 [95% CI 0.14, 0.69]; *p* = 0.004; Fig. 2). The pattern was similar for TIA and for the combined event of stroke and TIA (Table 1).

**Table 1:** Number of participants (referring to first event) and HR with 95% CI of primary and secondary outcomes

Outcome	Intensive therapy (*n*)	Conventional therapy (*n*)	HR (95% CI)	*p* value
Stroke	9	21	0.31 (0.14, 0.69)	0.004
TIA	1	9	0.08 (0.01, 0.66)	0.019
Stroke or TIA	10	25	0.28 (0.13, 0.58)	<0.001
Stroke + CVD death	18	37	0.36 (0.20, 0.63)	<0.001
Stroke + all-cause death	40	61	0.46 (0.31, 0.69)	<0.001
>1 stroke	1	10	–	0.018^a^

^a^*p* value calculated using Fisher’s exact test

We also demonstrated that intensive therapy resulted in a significantly reduced risk of the combined endpoint of stroke and CVD mortality (*n* = 55; HR 0.36 [95% CI 0.20, 0.63]; *p* < 0.001) and for the combined endpoint of stroke and all-cause mortality (*n* = 101; HR 0.46 [95% CI 0.31, 0.69; *p* < 0.001) (Table 1). The median time to stroke or death was 19.9 years in the intensive-therapy group and 11.4 years in the conventional-therapy group.

In the intensive-therapy group, 4 (11%) out of the 35 participants with a cardiovascular event experienced a stroke as their first manifestation of CVD, whereas this was the case in 14 out of 51 individuals (27%) in the conventional-therapy group.

Of the total 39 strokes evaluated in the study, 35 were ischaemic. The remaining four were haemorrhagic strokes, two of which were the first stroke in one patient in each of the two treatment groups, with the last two being a recurrent stroke occurring in two different individuals in the conventionally treated group.

Individuals who were originally randomised to conventional treatment were more likely to experience recurrent strokes than those originally randomised to intensified therapy, with one participant in the intensive-therapy group vs ten participants in the conventional-therapy group experiencing more than one stroke (*p* = 0.018).

A similar proportion of individuals in the two groups who experienced a stroke died during follow-up (78% in the conventional-therapy group vs 71% in the intensive-therapy group). However, participants in the original intensive-therapy group survived for a significantly shorter period after a stroke than those originally randomised to conventional therapy (median 0.3 years vs 3.1 years, respectively). This pattern was not observed for cardiovascular events not related to strokes (myocardial infarctions, amputations and peripheral or cardiac revascularisation), for which a smaller proportion of individuals randomised to intensive treatment vs conventional treatment died during follow-up after an event (55% vs 82%), with no difference in survival time after the evident event (median 3.4 years vs 3.9 years).

## Discussion

In the present post hoc analysis of data from individuals with type 2 diabetes and microalbuminuria from the Steno-2 Study with 21.2 years of follow-up, the hazard for first stroke after randomisation was reduced by 69% and the absolute risk by 15% with intensive therapy vs conventional treatment. Similarly, individuals originally randomised to conventional treatment were more likely to have recurrent strokes than those originally randomised to intensified therapy. Our findings once again emphasise the importance of an intensified multifaceted intervention approach, according to present EASD/ADA guidelines, for reducing CVD in patients with type 2 diabetes.

The proportion of individuals with a stroke during the follow-up period was 11% and 26% in the intensive- and conventional-therapy group, respectively. Since all participants in the Steno-2 Study had microalbuminuria (a marker of general vascular damage, indicating a higher risk for future complications) at baseline, the high incidence of stroke emphasises the importance of early screening for vascular risk factors in individuals with type 2 diabetes.

A surprising finding in the present post hoc analysis was the significantly shorter median lifespan after the first stroke of 0.3 years in the intensive-therapy group vs 3.1 years in the conventional-therapy group. We have no conclusive explanation for this finding. However, since the treatment algorithm for intensive therapy recommended a daily intake of 150 mg acetylsalicylic acid (ASA), it is reassuring that there was no increase in the number of cerebral haemorrhage events in those originally assigned to the intensive-treatment group.

In diabetic nephropathy, the classic Kimmelstiel–Wilson lesions are nodules of hyaline material in the glomerulus that occur due to an increase in mesangial matrix deposition [6]. In the brain, it has been demonstrated that the small arteries and arterioles that supply the territory of lacunar infarcts show segmental arterial disorganisation, fibrinoid degeneration and lipohyalinosis in those with diabetes or hypertension [7]. We have previously shown marked risk reductions for small-vessel disease in terms of progression of diabetic nephropathy with the intensified treatment approach used in the Steno-2 Study [3, 5]. It could, therefore, be hypothesised that this approach mainly prevents smaller lacunar infarctions associated with cerebral small-vessel disease, while major thromboembolic strokes with more severe symptoms and a poorer prognosis are prevented to a lesser extent.

As previously mentioned, it has been reported that the risk for stroke in individuals with type 2 diabetes has declined on a national scale [2]. However, despite intensified treatment as per the protocol in the Steno-2 Study, a residual risk for stroke of more than 10% over the duration of the ~20 year follow-up was observed. Also, due to the increasing number of individuals diagnosed with diabetes and the increased lifespan of these patients, stroke should still be considered a major personal and societal burden.

### Strengths and limitations

The major strength in the Steno-2 Study is the completeness of data for individuals enrolled in the trial. Except for one individual who emigrated from Denmark, leading to a lack of data during the observational part of the trial, a complete track of all endpoints and hospital admissions was available from Danish National Registers and applied in all analyses.

One major limitation of the study is the small sample size of 160 individuals. Another limitation is that a high-risk group (individuals with type 2 diabetes and microalbuminuria) were selected as the study population. Therefore, the magnitude of risk reduction we demonstrate here might not be attributable to a lower risk population. However, the risk reductions with intensive therapy presented in the current post hoc analysis are in coherence with previous cardiovascular risk reductions reported in the trial, both during its interventional and observational phase. Furthermore, since all participants were offered intensive treatment after the randomised phase, the effects presented may actually be an underestimation of the true effects.

## Conclusion

Our study demonstrates that stroke is a frequent and fatal complication in individuals with type 2 diabetes and microalbuminuria. An intensified multifactorial intervention significantly reduced the occurrence of this outcome, as well as the number of recurrent cerebrovascular events.

## Electronic supplementary material

ESM (PDF 75 kb)

## Data Availability

Data are available upon reasonable request to the corresponding author.

## Abbreviations

CVD: Cardiovascular disease
TIA: Transient ischaemic attacks

## References

[1] The GBD 2016 Lifetime Risk of Stroke Collaborators (2018). Global, regional, and country-specific lifetime risks of stroke, 1990 and 2016. N Engl J Med.

[2] Gregg EW, Li Y, Wang J (2014). Changes in diabetes-related complications in the United States, 1990–2010. N Engl J Med.

[3] Gæde P, Oellgaard J, Carstensen B (2016). Years of life gained by multifactorial intervention in patients with type 2 diabetes mellitus and microalbuminuria: 21 years follow-up on the Steno-2 randomised trial. Diabetologia.

[4] Gæde J, Oellgaard J, Ibsen R (2019). A cost analysis of intensified vs conventional multifactorial therapy in individuals with type 2 diabetes: a post hoc analysis of the Steno-2 study. Diabetologia.

[5] Gæde PH (2006). Intensified multifactorial intervention in patients with type 2 diabetes and microalbuminuria: rationale and effect on late-diabetic complications. Dan Med Bull.

[6] Kimmelstiel P, Wilson C (1936). Intercapillary lesions in the glomeruli of the kidney. Am J Pathol.

[7] Caplan LR (2015). Lacunar infarction and small vessel disease: pathology and pathophysiology. J Stroke.

[8] Oellgaard J, Gæde P, Rossing P (2018). Reduced risk of heart failure with intensified multifactorial intervention in individuals with type 2 diabetes and microalbuminuria: 21 years of follow-up in the randomised Steno-2 study. Diabetologia.

