# Supplementary material for: Beneficial impact of intensified multifactorial intervention on risk of stroke: outcome of 21 years of follow-up in the randomised Steno-2 Study
Source: Diabetologia. 2019 Jun 1;62(9):1575–80. doi: 10.1007/s00125-019-4920-3 (PMC6677690; doi:10.1007/s00125-019-4920-3)
Supplement: Supplementary file 1 — (PDF 75 kb) [file 125_2019_4920_MOESM1_ESM.pdf]

# Beneficial impact of intensified multifactorial intervention on risk of stroke: outcome of 21 years of follow-up in the randomised Steno-2 Study

## Electronic supplementary material

**ESM Methods**

Endpoint definitions:

All cardiovascular disease (CVD) endpoints in the Steno-2 Study were defined *a priory* and has been described in detail elsewhere (12). For the present analysis, the major definitions used were as described below:

Stroke

1) Definite ischaemic stroke: a computed tomography (CT) or magnetic resonance imaging (MRI) scan within 2 weeks of onset of a definite stroke (focal neurological deficit greater than 24 hours) with evidence of infarction, or autopsy confirmation.

2) Definite haemorrhagic stroke (primary intracerebral, subarachnoid, or secondary to cerebral infarction): confirmation with a CT or MRI scan within 2 weeks of stroke, or at autopsy or by lumbar puncture.

3) Stroke of unknown aetiology: definite stroke of unknown aetiology when CT, MRI or autopsy are not done, or where CT or MRI scan does not reveal pathology.

4) Non-fatal stroke post cardiovascular invasive interventions: stroke (as defined in 1, 2 or 3) associated to the intervention within 30 days of cardiovascular surgery, or within 7 days of cardiac catheterisation, or angioplasty, atherectomy, stent deployment or other invasive coronary or peripheral vascular interventions.

5) Non-fatal stroke post non-cardiovascular surgery: stroke (as defined in 1, 2 or 3) occurring within 30 days of noncardiovascular surgery.

Transient ischaemic attacks (TIA)

1) Definite TIA: focal neurological deficits with duration of less than 24 hours. Deficits must be observed and described by a physician.

2) Probable TIA: focal neurological deficits with duration of less than 24 hours. Deficits not observed or described by a physician.

3) TIA post cardiovascular invasive interventions: TIA (as defined in 1 or 2) associated to the intervention within 30 days of cardiovascular surgery, or within 7 days of cardiac catheterisation,

or angioplasty, atherectomy, stent deployment or other invasive coronary or peripheral vascular interventions.

4) TIA post non-cardiovascular surgery: TIA (as defined in 1 or 2) occurring within 30 days of non-cardiovascular surgery.

All possible cases were adjudicated for endpoints by an external committee masked for patients' original treatment allocation. Only definite events are included. Although not a requirement for the diagnosis of stroke or TIA as defined above, all patients with cerebral events in the Steno-2 Study had a CT or MRI scan performed.

**ESM Table 1**
Baseline values and baseline risk factors

| | Baseline values | | Hazard ratio 95% CI) | P-value |
|---|---|---|---|---|
| | Intensive | Conventional | | |
| Age (yr) | 55.0 ± 7,1 | 55.3 ± 7,2 | 1.008 (0.937, 1.083) | 0.84 |
| Sex (male) (n) | 63 | 56 | 2.048 (0.582, 7.210) | 0.26 |
| Systolic BP (mmHg) | 146 ± 20 | 149 ± 19 | 1.037 (1.013, 1,062) | 0.002 |
| HbA1c (mmol/mol) | 68 ± 6 | 73 ± 5 | 1.004 (0.980, 1.029) | 0.74 |
| HbA1c (%) | 8.4 ± 2.7 | 8.8 ± 2.6 | 1.044 (0.802, 1.367) | 0.74 |
| DM duration (yr) | 6.1 ± 5.8 | 7.9 ± 6.3 | 0.969 (0.886, 1.059) | 0.49 |
| Current smoking (n) | 32 | 27 | 0.729 (0.417, 1.275) | 0.27 |
| Known atrial fibrillation (n) | 3 | 4 | 1.249 (0.214, 7.276) | 0.81 |
| Fasting plasma total-cholesterol (mmol/l) | 5.4 ± 1,1 | 5.8 ± 1,3 | 1.056 (0.762, 1.464) | 0.74 |
| Glomerular filtration rate (ml/min/1,73 m2) | 116 ± 24 | 118 ± 25 | 0.984 (0.964, 1.004) | 0.11 |
| BMI (kg/m2) | 29.7 ± 3,8 | 29.9 ± 4,9 | 0.998 (0.893, 1.093) | 0.81 |
| Albumin excretion rate (mg/24 hour)* | 78 (61–120) | 69 (47–113) | 1.002 (0.996, 1.008) | 0.54 |
| Left ventricular ejection fraction (%) | 67.5 ± 8.3 | 67.5 ± 8.3 | 0.986 (0.935, 1.039) | 0.59 |
| Fasting plasma NT-ProBNP (pmol/l) | 35 (12-71) | 32 (13-67) | 1.001 (0.998, 1.004) | 0.61 |
| Known retinopathy (n) | 25 | 27 | 1.358 (0.540, 3.414) | 0.52 |

The left part of the table shows baseline characteristics for the two treatment groups.
The right part shows hazard ratios for a one unit increase in the variable of interest for continuous variables. For categorical variables the hazard ratio refers to male versus females, current smoking versus no or previous smoking, atrial fibrillation versus no atrial fibrillation, retinopathy versus no retinopathy, respectively. The p-value refers to the hazard ratio.

Intensive refers to the originally intensive-therapy group; Conventional refers to the originally conventional-therapy group.

In determination of baseline risk factors, we used Cox regression of the primary outcome with backwards elimination ($p < 0.10$) of the following variables: age, sex, known diabetes duration, smoking status, left ventricular ejection fraction, heart rate variability, known atrial fibrillation, BMI, systolic BP, glomerular filtration rate (GFR), retinopathy, HbA1c, plasma values of NT-proBNP and total cholesterol, and urinary albumin excretion rate (uAER).

Unless otherwise noted data are mean ± SD or median (interquartile range)
*Geometric mean (interquartile range)
